# Where does the fluid go?

**DOI:** 10.1186/s13613-025-01579-0

**Published:** 2025-10-14

**Authors:** Robert G. Hahn

**Affiliations:** Karolinska Institutet at Danderyds Hospital (KIDS), Stockholm, 171 77 Sweden

**Keywords:** Plasma volume, Cardiac damage, Crystalloid fluid, Pharmacokinetics

## Abstract

**Background:**

Liberal administration of crystalloid fluid is often required to maintain adequate tissue perfusion when treating life-threatening conditions. Current knowledge indicates that either overhydration or underhydration can promote complications. This review describes how fluid distributes between body compartments, with the aim of finding insights into pathophysiological mechanisms that can explain why fluid overload may cause complications and even be fatal.

**Main text:**

The skin, intestinal wall, and lungs are known primary locations of excess amounts of crystalloid fluid in humans. Microscopic studies in animals show that infusion of > 100 mL/kg of crystalloid fluid also causes interstitial dilatation and swelling of the heart, tissue breakup, and cardiac hypoxia. Volume kinetic analysis has identified several factors that promote peripheral edema during general anesthesia. Volume kinetics also shows that increasing volumes of crystalloid fluid sequentially expands three body fluid compartments: the plasma, a fast-exchange interstitial volume, and a more remote slow-exchange interstitial volume (in scientific jargon called “the third fluid space”). In settings of overhydration, the slow-exchange space operates as an overflow reservoir and quickly begins to accumulate fluid when the fast-exchange compartment has increased by 600–800 mL, which corresponds to infusing approximately 1.3–1.5 L of crystalloid fluid into the plasma over 30 min. Apart from overhydration, accumulation of fluid in the slow-exchange space occurs in inflammatory conditions, whereby cytokines and vasoactive molecules create a suction pressure that withdraws fluid from the fast-exchange space. This suction decreases lymphatic flow, causing hypovolemia and hypoalbuminemia in addition to peripheral edema. Preeclampsia and sepsis are examples of this complex kinetic situation. Albumin (20%), a hyper-oncotic colloid, might be used to modify peripheral edema by recruiting interstitial (lymphatic) fluid and stimulating diuresis.

**Conclusion:**

Excess amounts of crystalloid fluid accumulate in body regions, such as the skin and intestinal walls, that have a high compliance for volume expansion. The heart is potentially a key trouble spot in severe overhydration. Accumulation of fluid in an interstitial fluid space that equilibrates slowly with the plasma volume occurs in settings of overhydration and inflammation. Pathophysiological mechanisms that explain the complications and fatal outcomes of overhydration are insufficiently known in humans.

## Introduction

Fluid administration is an integral part of treatment in the intensive care unit (ICU). Water loss by evaporation (≈700 mL/day) must be compensated by administering fluid if the patient is unable to, or not allowed to, eat and drink [1]. Fluid administration should also ensure a urine flow that exceeds 0.5 mL/min to allow metabolic end products to be fully excreted. Importantly, severe disease involves redistribution of blood flows due to blunting of the adrenergic tonus and subsequent vasodilatation. This redistribution, in addition to possible disease-specific toxic effects, requires that the plasma volume (PV) be expanded with an electrolyte-based crystalloid (first choice) or colloid (secondary choice) fluid to maintain adequate circulation and preserve organ function [2]. 

Fluid resuscitation and optimization of severely ill patients typically requires PV expansion for 1–3 days, followed by withdrawal of excess body fluid (“de-resuscitation”) when the medical condition improves [3, 4]. Notably, body fluid volumes may increase considerably during this period. The volume loading is not innocuous, as shown by the many ICU studies that associate positive fluid balance with increased mortality [5, 6]. Furthermore, liberal fluid administration during surgery might induce complications that prolong hospital stay or require ICU care,^7^ where continued liberal fluid administration during the postoperative period adds to the harm. A study by Brismar et al. associated a 50% mortality rate with a positive fluid balance of 10–18 L during the first week in patients who developed acute respiratory distress syndrome (ARDS) after surgery [8]. 

A reasonable question to ask is: Where does excess fluid go?

### Observations in humans

Crystalloid fluid is said to equilibrate throughout the extravascular space, but the distribution of the excess volume depends on how easily the local tissues expand. The interstitial volume amounts to 50% of the skin mass but accounts for only 10% of muscle; moreover, the compliance for volume expansion is twice as high in the skin compared to muscle [9]. Therefore, most of the excess fluid ends up in the skin where it may impair wound healing and exacerbate local infection [7]. By contrast, bone tissue does not undergo volume expansion at all; therefore, no accumulation of excess fluid occurs in bone and only a minimal amount in body organs surrounded by a tight capsule.

The lungs are another location where excess fluid is found. Volume overload promotes the development of pulmonary edema [7] and ARDS, [8] which can occur with a delay of several days and even be fatal [10]. The edema is governed by the balance between capillary filtration and lymphatic flow and, importantly, the lymphatic drainage of the thorax and abdomen via the thoracic duct is greatly reduced by mechanical ventilation [11]. 

The gastrointestinal wall is the third site for the accumulation of excess fluid. The gut mucosa has fenestrated capillaries that allow for fast transfer of fluid and solutes. Animals [12] and humans [13] alike accumulate more water in the gastrointestinal wall when infused with crystalloid rather than colloid fluid.

These three areas correlate well with known locations for acknowledged edema-associated complications. For example, administration of >2 L of crystalloid prolonged postoperative gastrointestinal recovery by several days,[14,15] while infusing 6 L instead of 3 L of crystalloid during colon surgery increased the risk of wound infection, suture insufficiency, bleeding, and pneumonia [7]. Similarly, rapid administration of 2 L of Ringer’s over 15 min in young women caused acute swelling sensations in the face and arms, as well as slight dyspnea. These symptoms did not occur when the infusion time was doubled, possibly due to the tissues having more time to adapt to the volume changes [16]. 

### Morphological changes

Extreme fluid overload causes morphological organ damage and may even be fatal. The subendocardium of the heart was identified as a key trouble spot when isotonic saline was included in a series of animal studies on irrigating solutions [17–19]. The heart area is sensitive to hypoxic events, as it is rich in fibers belonging to the electrical conduction system. Thus, disturbances in cardiac rhythm may occur if the oxygen supply becomes insufficient. Lesions are often found in the subendicardium of humans who die suddenly [20]. 

Specifically, in rabbits, the experimental infusion of 100 mL/kg of saline over 30 min caused a modest weight increase in the heart but clear interstitial dilatation and swelling [17]. Immunohistochemistry showed hypoxic lesions in the left ventricle and the papillary muscles. The renal collecting ducts were swollen, and excessive fluid vacuoles appeared in the choroid plexus, but no brain edema or swelling of the hepatocytes was noted.

**Fig. 1:**
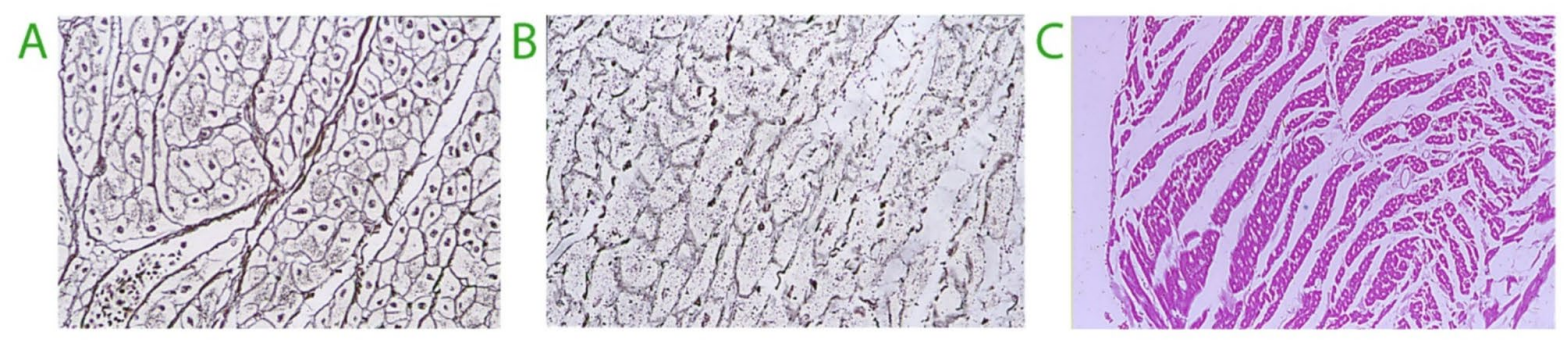
**A** Normal mouse heart stained to visualize the cytoarchitecture. **B** Damaged cytoarchitecture of the heart in a mouse that died after receiving 300 mL/kg of isotonic saline over 60 min via the tail vein. Light microscopy 600×, Gordin and Sweet’s silver staining of reticular fibers. **C** Interstitial pools of free fluid in the myocardium of a rabbit 2 h after receiving 100 mL/kg of fluid containing 1.5% glycine. Photos taken in the framework of studies 17 and 18

In mice, hypoxic changes in the heart were detected in 21% of the animals receiving 200 mL/kg of saline and in 33% of animals who were given 300 mL/kg over 60 min [18]. Lesions of the histoskeleton were found in 31%, in correlation with the occurrence of hypoxic lesions. Interstitial dilatation was common. Mortality was 30% for both doses of saline **(**Fig. 1**)** [19]. Despite the demonstration of infusion-related lesions in experimental animals, the occurrence of morphological lesions in overhydrated humans remains poorly documented.

### General anesthesia

Many patients in the ICU are anesthetized and mechanically ventilated. The differences in fluid distribution between the anesthetized and awake states have been studied using volume kinetics, which provides a non-anatomical whole-body view of how the body handles infusion fluids [21]. 

General anesthesia inhibits the diuretic response to PV expansion by 85–90%, [22] mostly due to the accompanying reduction in the arterial pressure, [23] with the inhibition transmitted via renal sympathetic fibers [24]. 

Additionally, volatile and intravenous agents inhibit lymphatic pumping, thereby slowing down the return flow of distributed fluid to the plasma [25]. As mentioned previously, lymphatic flow of the thorax and abdomen is reduced by mechanical ventilation [11]. 

Finally, despite the lower arterial pressure, capillary filtration accelerates [26]. This effect is probably due to the general reduction of the centrally controlled adrenergic tonus, which causes arteriolar vasodilatation and relaxes the pre-capillary sphincters. These sphincters are small rings of smooth muscle that regulate the blood flow into the capillaries. The resulting facilitation of the blood flow increases the capillary pressure, which promotes filtration. Relaxation of the sphincters also open previously closed capillaries, [27] which increases the number of perfused vessels [28] and thereby the surface area available for transcapillary filtration. This proposed facilitating effect of a reduction of the centrally controlled adrenergic tonus on capillary filtration was confirmed in experimental spinal anesthesia where the capillary leakage of fluid occurred faster in anesthetized body regions (the legs) than in non-anesthetized areas (the arms) as soon as the arterial pressure had decreased as a sign of an effective blockade [29]. 

The summary effect of these three changes of general anesthesia on fluid kinetics is greater fluid retention in the whole body; however, the increase in interstitial volume is disproportionately greater than in the PV [30]. Hence, general anesthesia creates peripheral edema, but, fortunately, the kinetic alterations are normalized soon after the patient awakens [31, 32]. 

### Three fluid compartments

Crystalloid fluid administered by intravenous infusion is first distributed throughout the plasma volume (approximately 3 L) and then equilibrates with the expandable part of the interstitial space (normally 6–8 L) during a period of 30 min [21]. The interstitial space consists of a collagen fiber meshwork, a gel phase of glycosaminoglycans (GAGs, mostly hyaluronan), and a salt solution with plasma proteins [33]. The relative composition of these constituents varies greatly between tissues. Morphological studies show that the interstitial space harbors fluid in a “free” phase and that fluid is also present in the gel phase [34]. Free fluid is transported in channels that allow long-distance movement [35, 36] while the dense collagen bundles of the gel phase markedly restrict fluid movement (low hydraulic conductivity) [9, 33]. The content of protein and water and their mobility in the gel is greatly affected by the presence of the body cells, stiffness of the gel, and exclusion effects due to the volume and electrical charges of the macromolecules embedded in the meshwork [33]. Interstitial fluid is returned to the plasma via lymphatic vessels that begin with free endings in the interstitium. Collecting lymphatic vessels have valves to prevent retrograde flow and are endowed with smooth muscle that have pumping capacity [25]. 

**Fig. 2:**
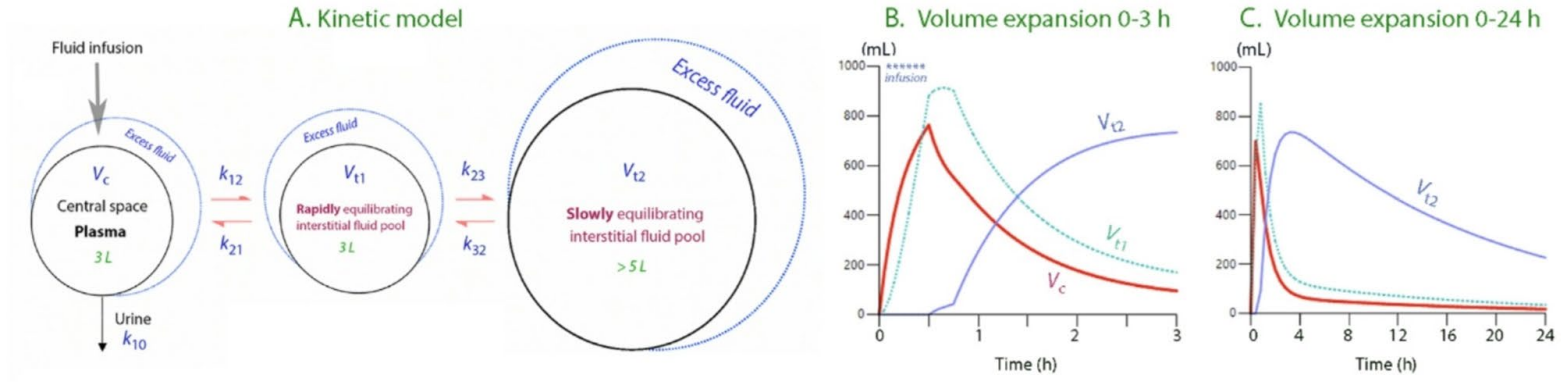
**A** Relationship between *k*_21_ (return flow to the plasma) and *k*_23_ (fluid to “third space”) shown in the three-volume kinetic model that is valid for infusion volumes > 1.5 L. **B** Simulation of fluid distribution based on kinetic analysis of 100 awake volunteers receiving an average of 1.9 L of Ringer’s solution over 30 min; the volunteers were monitored for 4 h. The value of *k*_23_ was estimated by covariate analysis for each 5-min period between 15 and 45 min since the administration of Ringer´s was initiated to determine precisely when *V*_t2_ opened for volume accumulation. **C** Same plot as (B) but the fluid distribution is predicted up to 24 h

Volume kinetic analysis confirms that the human body contains three extracellular fluid compartments that become expanded by electrolyte-based crystalloid fluid (Fig. 2A). Small volumes (250–400 mL) are distributed only in the plasma, whereas intermediate volumes (up to 1.3 L) are also distributed in a fast-exchange interstitial fluid space (*V*_t1_) [37]. Hence, a minimum dilutional decrease in the colloid osmotic pressure and an increase in the intravascular hydrostatic pressure are required to distribute excess fluid to the interstitium. *V*_t1_ is a functional space but it is believed to correspond grossly to the sum of the interstitial “free fluid” phase and the lymphatic vessels.

Large volumes of fluid are also distributed to a slow-exchange interstitial space (*V*_t2_), which apparently operates as a fluid reservoir that prevents intravascular fluid overload. *V*_t2_ opens up for volume expansion when *V*_t1_ has expanded by 600–800 mL, which corresponds to a relatively fast (30 min) intravenous infusion of 1.3–1.5 L of fluid [37]. Fluid accumulated in *V*_t2_ has a very slow turnover and, therefore, most of the infused volume (that which has not been excreted) resides in *V*_t2_ at 2 h after an infusion ends (Fig. 2B).

The opening up of *V*_t2_ for fluid accumulation is sudden and likely corresponds to the point at which fluid administration has raised the interstitial hydrostatic pressure to zero from its normal sub-atmospheric range of -2 to -4 mmHg. In the 1960s, Guyton et al. showed that the interstitial compliance for volume expansion increases dramatically (×100,000) when the zero pressure (i.e., the same pressure as the surrounding air) is exceeded [38, 39]. Before this point is reached, water molecules probably pass into *V*_t2_ but are quickly exchanged for other water molecules because the compliance for volume expansion is too low.

### Physiology of the slow-exchange fluid space

Fluid accumulation in *V*_t2_ is likely to be of paramount importance in the “fluid overload” problem occurring in patients admitted to the ICU. On the one hand, *V*_t2_ apparently offers protection against intravascular fluid overload; on the other hand, the turnover of fluid in this compartment is very slow, which greatly prolongs the persistence of excess fluid in the body (Fig. 2C) [37]. The slow turnover of fluid accumulated in *V*_t2_ makes it understandable that the “de-resuscitation phase” in patients in the ICU might require several days, during which peripheral edema co-exists with hypovolemia. It also explains why the body weight increase after major surgery can persist for up to one week [7, 40]. 

*V*_t2_ is a functional/physiological entity that is probably spread throughout the physiological interstitium and likely represents the gel phase, as fluid movement is known to be restricted there. When zero pressure is exceeded, the sub-atmospheric pressure no longer holds the tissues together, and scattered pools of fluid appear between the cell bundles. This is noted in the skin as “pitting edema”, but it can also appear elsewhere, such as in the heart muscle (Fig. 1C).

Fluid equilibration between plasma and *V*_t1_ is quite efficient, but equilibration between *V*_t2_ and *V*_t1_ is not. Another characteristic is that *V*_t2_ opens but clearly does not close at a specific “trigger pressure” in *V*_t1_. Fluid accumulated in *V*_t2_ must first find its way back to *V*_t1_, which may not be straightforward due to the complicated structure of the gel meshwork.

Restoration of the sub-atmospheric pressure of the interstitium is probably required for *V*_t2_ to finally close. The sub-atmospheric pressure is explained by a state of dehydration that is created and maintained by tensile forces due to cell-collagen interactions. The interstitium has high metabolic activity, [9] but full restoration of *V*_t2_ still seems to require days for full completion. Effective lymphatic flow coupled with slow capillary filtration is also required for a sub-atmospheric pressure to be maintained [33]. 

### Manipulating the slow-exchange space

An important question is whether *V*_t2_ can be manipulated in settings of overhydration. Opening of *V*_t2_ can be prevented by infusing less fluid and by providing the fluid more slowly, at least in a patient in the awake state [37]. This is because the kidneys then have more time to excrete the excess volume. Another possible approach for preventing *V*_t2_ opening is to reduce the interstitial fluid pressure, which occurs in response to blood loss and infusion of hyper-oncotic colloid (dextran 40) [41]. *V*_t2_ opening requires a greater expansion of *V*_t1_ during general anesthesia (approximately 950 mL) [37] due to hypotension-derived redistribution of >200 mL of the interstitial fluid to the plasma [42, 43]. The interstitial hydrostatic pressure might decrease due to acidosis, [9] but this has not yet been confirmed to change the “trigger” for opening of *V*_t2_. Adrenergic stimulation by using drugs increases (β_1_-receptors) or decreases (α_1_-receptors) the expansion of *V*_t1_, [30] suggesting that the filling of *V*_t2_ would probably become affected as well. Less is certain about how to dehydrate *V*_t2_ once it is filled. Preliminary data suggest that high plasma aldosterone accelerates this process, but this finding requires further confirmation.

A way to reduce the total body edema is by manual compression, which has been evaluated in septic patients [44]. Manual compression also increased cardiac preload and reduced the wrist circumference in intensive care patients [45]. An in vitro parallel to these observations is that counter-pressure prevents the swelling that most tissues undergo if excised and placed in a bath of isotonic saline [46]. Hence, the interstitium is normally under a constant state of dehydration and absorbs water if the dehydrating mechanisms are overwhelmed or disrupted. Moreover, increased cardiac preload probably increases the urine output, which reduces the positive fluid balance that is typical for the first days of ICU care.

### The slow-exchange space in inflammation

Inflammation adds a dimension to the expansion of *V*_t2_ that is closely associated with the well-known triad of hypovolemia, hypoalbuminemia, and peripheral edema. As argued elsewhere, [28] this triad, which is often seen in ICU patients, is likely due to molecular interactions in *V*_t2_ caused by pro-inflammatory cytokines and other signal substances, such as TNF-alpha, IL-1-beta, IL-6, and lipopolysaccharide [47]. Some of them diffuse to the interstitium from the plasma (for example, TNF-alpha) while most pro-inflammatory cytokines are produced locally (such as IL-1-beta) [48]. These molecules reverse integrin-collagen bindings, thereby decreasing the interstitial pressure and creating a “suctioning” effect in the tissue [47–51]. Platelet-derived growth factor might counteract this process, [52,53] which is a generally accepted mechanism for the formation of edema [31]. In acute burn injury cases, the interstitial pressure can be reduced from − 2 to -50 mmHg, [49,50] whereas in severe inflammation cases, the pressure drop might end at -10 mmHg [33, 51, 54]. For a detailed discussion, see the excellent review by Dargent et al.. {55]

### Capillary leakage versus accumulation in V_t2_

Reed and Rubin [51] have proposed that the increased negative interstitial pressure because of generalized inflammation can explain the observed increase in transcapillary leakage without assuming a change in capillary permeability. However, as discussed below, whether a change in capillary permeability alone can explain the peripheral edema, hypovolemia, and hypoalbuminemia seen in inflammatory conditions remains questionable.

Sepsis accelerates the capillary leakage of albumin from approximately 5% to 7% of the plasma pool per hour, [56] which is not dramatically different from the 5.3% found after major abdominal surgery [57]. Significantly accelerated capillary leakage is a hallmark of the rare idiopathic capillary leak syndrome, but the modest changes encountered in the event of sepsis and after major surgery are likely compensated by an increased lymphatic flow in the absence of other pathophysiological factors. Hence, the flow in the thoracic duct increases a few minutes after volume loading with crystalloid fluid, [58] illustrating the well-established linear relationship between interstitial fluid volume and lymphatic flow [9]. 

In healthy adults, full equilibration between the plasma and *V*_t1_ is achieved (i.e., the dilution is the same) within 30 min after ending the infusion of even a large volume of crystalloid fluid [21, 26]. The compensatory increase in lymphatic flow due to increased transcapillary leakage, regardless of the reason, is called “interstitial washdown” and normally restores normovolemia and even increases the intravascular content of albumin [59, 60]. Therefore, studying disturbances of the lymphatic flow and accumulation of fluid in *V*_t2_ might be more fruitful approaches than focusing only on capillary leakage when exploring maldistribution of fluid in inflammatory disorders.

### Lymphatic flow versus filling of V_t2_

The physiological background of our understanding of why fluid accumulates in *V*_t2_ in response to overhydration is laid out by Guyton, [38,39] while the effects of inflammation have been explored by Reed, Wiig, and Lund [47–52]. However, their findings were only recently put into perspective in living humans using volume kinetics. Importantly, these kinetic analyses highlight the role of lymphatic flow in the maldistribution of fluid and albumin in conditions in which cytokines and other vasoactive and inflammatory molecules are likely to have decreased interstitial pressure. Examples are given below.

#### Preeclampsia

**Fig. 3:**
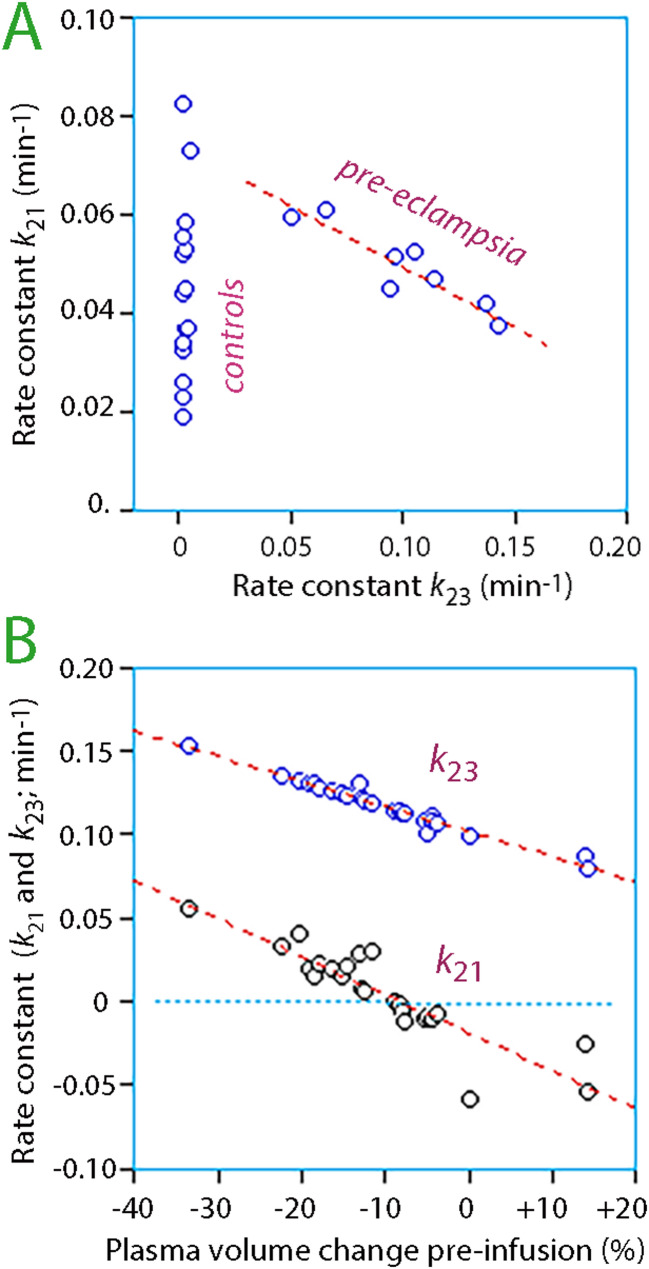
**A** Rate constant *k*_23_ (fluid to the “third space”) *versus k*_21_ (return flow to the plasma) in 8 women and 18 pregnant and nonpregnant women analyzed after receiving 12.5 mL/kg of Ringer´s solution over 30 min. Elevation of *k*_23_ was seen only in women with preeclampsia. Three women without preeclampsia had a higher k_21_ than 0.10 min^- [1]^ and are not shown in this figure. Data were obtained from ref. 61. **B** Rate constants *k*_23_ and *k*_21_ *versus* the constriction in plasma volume, which is calculated based on the hemoconcentration, occurring during 1 h between the induction of sepsis and the infusion of Ringer’s solution in 25 sheep. The data were obtained from ref. 63

In a study of mild to moderately severe preeclampsia, the rate constant for the return flow of fluid from *V*_t1_ to the plasma (*k*_21_) attained a very low value [61]. This suggests that the lymphatic flow was slow. Reanalysis of these data shows that the reduction is secondary to filling of *V*_t2_. Hence, the response appears to reflect competition between the two flows that move fluid away from *V*_t1_; a high *k*_23_ was associated with a lower *k*_21_ (Fig. 3A); however, due to the dehydrating effect of *k*_23_ on *V*_t1_, the product of *k*_21_ and the expansion of *V*_t1_, which is a quasi-measure of the lymphatic flow, was reduced by as much as 80% compared to the lymphatic flow observed in pregnant and nonpregnant women without preeclampsia. On the assumption that the fluid entering *V*_t2_ contains albumin, simulations suggest that poor lymphatic flow could reduce plasma albumin by as much as 50% in 24 h [30] although some of this decrease could be counteracted by increased albumin synthesis [62]. 

#### Sepsis

Reanalysis of data on 25 septic sheep [63] suggests that competition between fluid accumulation in *V*_t2_ and lymphatic flow can become a potentially serious issue. Here, sepsis was induced while Ringer’s solution was withheld for 1 h, during which period the PV contracted by an average of 10%. A strong contraction implied that more fluid was present in *V*_t1_ when infusion was initiated. However, the lack of contraction of the PV during that time jeopardized lymphatic flow, and *k*_21_ could even become negative (Fig. 3B). Just as observed with preeclampsia, the tendency to allocate fluid to *V*_t2_ was greater than the tendency to return distributed fluid to the plasma via *k*_21_, resulting in an abnormal *k*_21_/*k*_23_ ratio (the typical ratio is 2–3; here, it was 0.9). Provided that a high *k*_23_ holds down *k*_21_ and not the opposite, this relationship suggests that lymphatic flow could stagnate completely if crystalloid fluid is not administered at an early stage of sepsis. Thus, fluid loading in this setting alleviates hypovolemia and hypoalbuminemia, whereas peripheral edema worsens.

This kinetic analysis agrees with claims that the lymphatic flow is slight at interstitial pressures below − 6 mmHg, [59] which is the case is sepsis [33, 51, 54]. A contributing factor to the pathological scenario might also be that cytokine-induced nitric oxide production inhibits lymphatic pumping [25]. If used, mechanical ventilation [11] and anesthesia drugs [25] further impair the lymphatic flow.

### Maximum filling of V_t2_

**Fig. 4:**
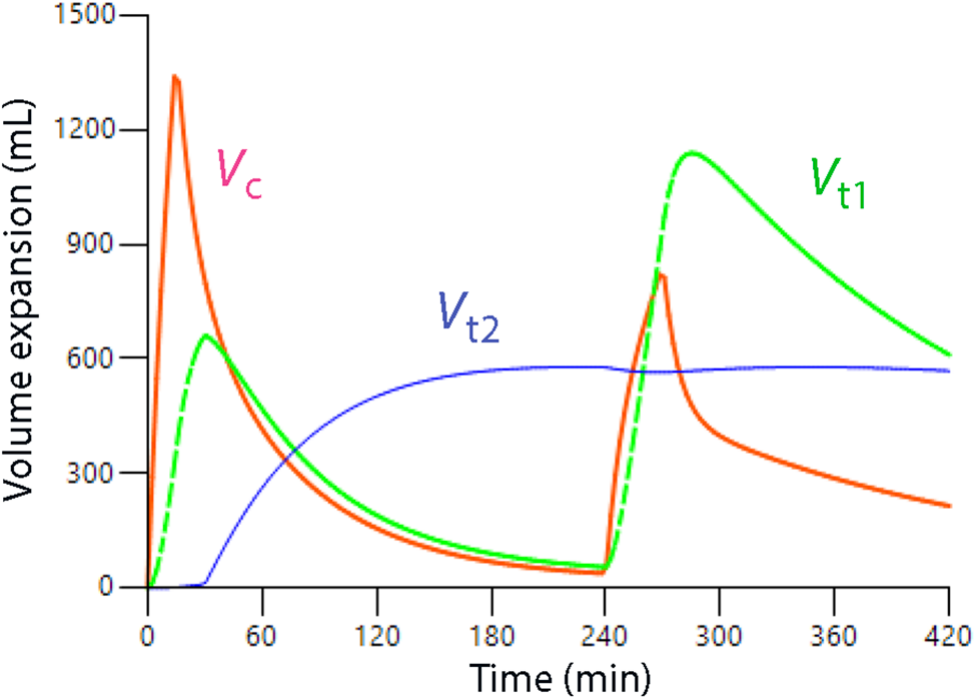
Simulation of the volume expansion of the three body fluid compartments that become expanded by crystalloid fluids given twice 4 h apartment. Ten male volunteers (mean body weight 81 kg) received 25 mL/kg of Ringer´s acetate over 15 min and later over 30 min. Note that the slow-exchange compartment (*V*_t2_) does not further expand during the second infusion. The infusions were also given in the reverse order in a cross-over fashion, which showed the same result. Kinetic re-analysis of data from Reference 62

The slow-exchange fluid compartment is not a bottomless pit where excess fluid can accumulate indefinitely. Re-analysis of experiments in healthy volunteers [64] where infusions were repeated after 4 hours suggest that acute filling of *V*_t2_ peaks at approximately 600 mL. Further volume loading expands *V*_t1_ (Fig. 4). The threshold might correspond to the hockey-stick-like decrease in compliance described by Aukland and Reed [9] to occur when the “interstitial hydration is excessive”. Importantly, stretching due to longstanding overhydration increases the compliance of both the vascular [65] and interstitial spaces [46] over time (”stress relaxation” or “delayed compliance”) which explains, for example, the very high compliance for local volume expansion in secondary lymphedema [33]. Therefore, *V*_t2_ is likely to gradually accommodate more fluid during an ICU stay.

Another example of maximum filling of *V*_t2_ comes from an experimental study in pigs where overhydration and inflammation co-occurred. An initial volume load first opened *V*_t2_ whereafter a septic event was induced [66]. The suction pressure had probably subsided 24 h later, as is the case in burns, [67] when a new infusion as given. The additional volume load only entered the *V*_t2_ if the total increase in body weight was < 15%. This suggests that filling of *V*_t2_ had a limit of 15% of the body weight 24 h after volume loading and a septic event, which is 5 times higher than the limit found after acute overhydration in the volunteers.

### Hyper-oncotic albumin

**Fig. 5:**
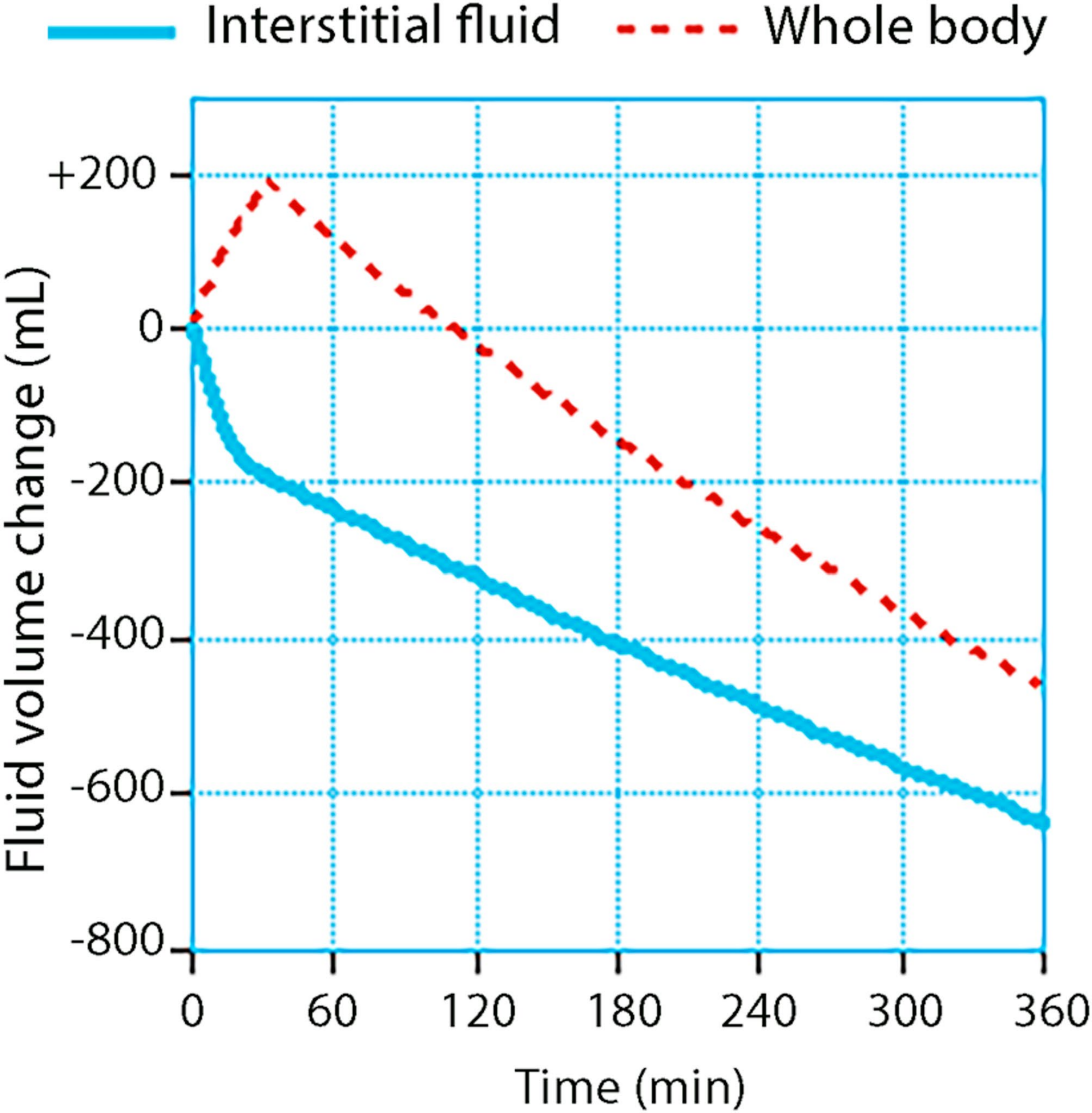
Recruitment of interstitial fluid and whole-body fluid balance during and after the infusion of 230 mL/kg of 20% albumin over 30 min. Volume kinetic data were obtained from 83 volunteers and clinical patients who received this fluid in different settings but using the same protocol (references 75–78)

The use of colloid fluids has been declining in perioperative care; [68] however, there has been an increase in the administration of albumin in ICUs [69]. Infusing hyper-oncotic albumin is a strategy aimed at reducing the need for large volumes of crystalloid fluid when PV expansion is needed, [70] although the capacity of albumin to allocate body fluid to the plasma has been questioned [71, 72]. Data obtained from healthy volunteers show that 20% albumin withdraws 3.4 times the infused volume from the interstitium within 5 h, [73] primarily via the lymphatic route [74]. Two-thirds of this volume is excreted as urine; hence, infusing 20% albumin increases the PV by twice the infused volume [75, 76]. Overall, the interstitium and the whole body are dehydrated (Fig. 5). The intravascular persistence time of the expanded volume is 5–7 h in healthy volunteers, [77,78] 10 h in postoperative patients, [79] and even longer in patients undergoing surgery, [80] which should be kept in mind when considering repeat infusions. The diuretic effect is well maintained during hypotension, which is not the case when diuresis is induced with crystalloid fluid [81]. Logically, the interstitial pressure is reduced, which is known to occur after infusion of dextran [40,41] and this would mean that the lymphatic flow will be stimulated and the pressure threshold for opening *V*_t2_ becomes more distant. However, the benefit with respect to interstitial edema is unclear if a crystalloid fluid is infused soon after a colloid because much of the crystalloid is then dislocated to the interstitium [82]. 

## Conclusion

An excess volume of crystalloid fluid ends up in body regions, such as the skin, intestinal wall, and lungs, where the compliance for volume expansion is high. Observations in animals suggest that overhydration causes tissue breakup and cardiac hypoxia. Recent insights into the physiology of an interstitial fluid space that equilibrates slowly with the plasma volume may provide a better understanding of the pathophysiology of fluid-related complications. The “slow exchange” interstitial space apparently operates as an overflow reservoir in settings of volume overload. In inflammatory conditions, withdrawal of distributed fluid into this space reduces lymphatic flow, which promotes hypovolemia and hypoalbuminemia.

## Data Availability

The original data related to the referenced original articles that RGH has authored or coauthored can be obtained at reasonable request by emailing robert.hahn@ki.se.
